# Diaqua­bis­{1-[(1*H*-benzimidazol-2-yl)meth­yl]-1*H*-imidazole-κ*N* ^3^}dichlorido­cadmium hexa­hydrate

**DOI:** 10.1107/S160053681202034X

**Published:** 2012-05-12

**Authors:** Xin-Nian Xie, Meng Lu, Juan Yuan, Huai-Xia Yang

**Affiliations:** a Pharmacy College, Henan University of Traditional Chinese Medicine, Zhengzhou 450008, People’s Republic of China

## Abstract

In the title complex, [CdCl_2_(C_11_H_10_N_4_)_2_(H_2_O)_2_]·6H_2_O, the Cd^II^ atom is located on a twofold rotation axis and is coordinated by two N atoms from two 1-[(1*H*-benzimidazol-2-yl)meth­yl]-1*H*-imidazole ligands and two water O atoms in equatorial positions and by two Cl atoms in axial positions, leading to an elongated octa­hedral environment. The two coordinating and two of the lattice water mol­ecules are also located on twofold rotation axes. In the crystal, complex mol­ecules and solvent water mol­ecules are linked through a complex inter­molecular N—H⋯O, O—H⋯N, O—H⋯O and O—H⋯Cl hydrogen-bonding scheme into a three-dimensional network.

## Related literature

For background information on Cd^II^ complexes constructed from *N*-heterocyclic ligands see: Jin *et al.* (2012 ▶); Liu *et al.* (2008 ▶).
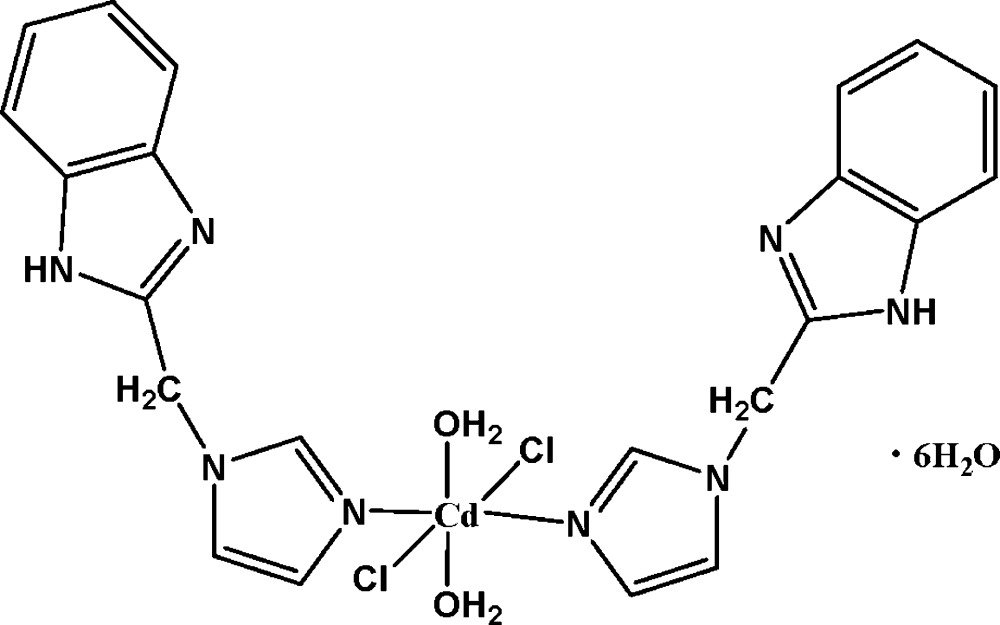


## Experimental

### 

#### Crystal data

[CdCl_2_(C_11_H_10_N_4_)_2_(H_2_O)_2_]·6H_2_O
*M* *_r_* = 723.89
Monoclinic, 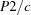

*a* = 8.3562 (17) Å
*b* = 10.236 (2) Å
*c* = 17.972 (4) Å
β = 98.80 (3)°
*V* = 1519.1 (5) Å^3^
*Z* = 2
Mo *K*α radiation
μ = 0.95 mm^−1^
*T* = 293 K
0.18 × 0.17 × 0.14 mm

#### Data collection

Rigaku Saturn diffractometer
Absorption correction: multi-scan (*CrystalClear*; Rigaku/MSC, 2004 ▶) *T*_min_ = 0.847, *T*_max_ = 0.878
18365 measured reflections
3632 independent reflections
3345 reflections with *I* > 2σ(*I*)
*R*_int_ = 0.044

#### Refinement

*R*[*F* ^2^ > 2σ(*F* ^2^)] = 0.047
*wR*(*F* ^2^) = 0.123
*S* = 1.00
3632 reflections
188 parameters
H-atom parameters constrained
Δρ_max_ = 0.66 e Å^−3^
Δρ_min_ = −0.63 e Å^−3^

### 

Data collection: *CrystalClear* (Rigaku/MSC, 2004 ▶); cell refinement: *CrystalClear*; data reduction: *CrystalClear*; program(s) used to solve structure: *SHELXS97* (Sheldrick, 2008 ▶); program(s) used to refine structure: *SHELXL97* (Sheldrick, 2008 ▶); molecular graphics: *SHELXTL* (Sheldrick, 2008 ▶); software used to prepare material for publication: *publCIF* (Westrip, 2010 ▶).

## Supplementary Material

Crystal structure: contains datablock(s) global, I. DOI: 10.1107/S160053681202034X/wm2627sup1.cif

Structure factors: contains datablock(s) I. DOI: 10.1107/S160053681202034X/wm2627Isup2.hkl

Additional supplementary materials:  crystallographic information; 3D view; checkCIF report

## Figures and Tables

**Table 1:** Selected bond lengths (Å)

Cd1—N1	2.289 (3)
Cd1—O2	2.349 (4)
Cd1—O1	2.362 (4)
Cd1—Cl1	2.6445 (13)

**Table 2:** Hydrogen-bond geometry (Å, °)

*D*—H⋯*A*	*D*—H	H⋯*A*	*D*⋯*A*	*D*—H⋯*A*
N3—H3*A*⋯O6	0.86	2.15	2.909 (4)	148
O2—H2*W*⋯O4	0.85	1.95	2.776 (3)	162
O4—H4*W*⋯N4	0.85	1.91	2.756 (4)	171
O4—H5*W*⋯O3	0.85	2.06	2.857 (4)	156
O3—H3*W*⋯Cl1^i^	0.85	2.41	3.234 (3)	165
O5—H6*W*⋯Cl1^i^	0.85	2.28	3.110 (3)	164
O1—H1*W*⋯O6^ii^	0.85	1.88	2.723 (4)	173
O6—H7*W*⋯O5^iii^	0.85	1.98	2.799 (4)	162
O6—H8*W*⋯O4^iv^	0.85	1.96	2.737 (4)	152

Symmetry codes: (i) 
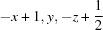
; (ii) 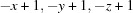; (iii) 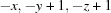; (iv) 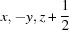.

**Fig. 1.**
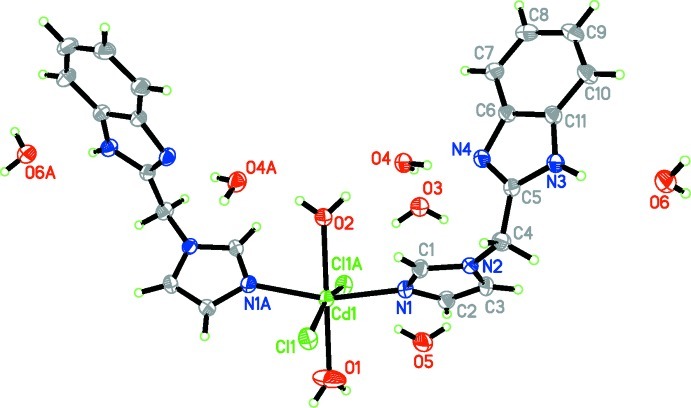
View of the title complex showing labeling and 30% probability displacement ellipsolids. [Symmetry code A: -x + 1, y, -z + 1/2.]

**Fig. 2.**
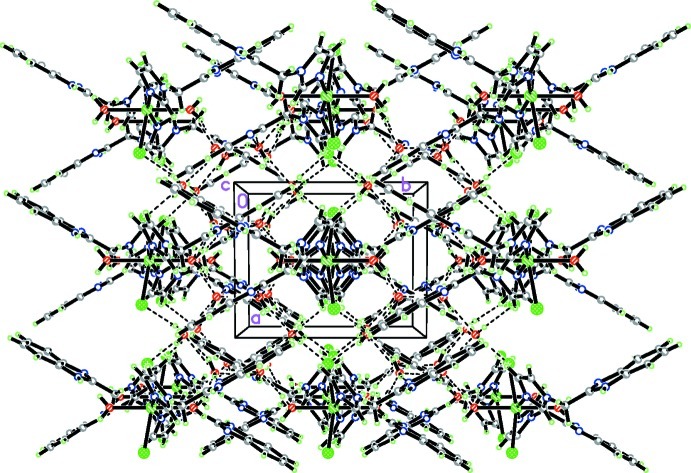
Packing plot of the title complex with hydrogen bonds indicated by dashed lines.

## supplementary crystallographic information

### Comment

A large number of Cd^II^ complexes constructed from *N*-heterocyclic ligands have been synthesized since the Cd^II^ ion is a useful building block or connecting node. Moreover, closed-shell *d*^10^—*d*^10^ Cd—Cd interactions can often give rise to supramolecular motifs and interesting properties (Jin *et al.*, 2012; Liu *et al.*, 2008). In order to further explore new such Cd-containing compounds and their structures, we selected 1-((1*H*-benzimidazol-1-yl)methyl)-1*H*-imidazole as a ligand to self-assembly with CdCl_2_ and obtained the title complex, {[CdCl_2_(C_11_H_10_N_4_)_2_(H_2_O)_2_](H_2_O)_6_}.

The Cd^II^ ion displays an elongated octahedral coordination environment defined by atoms N1, N1A from two monodentate 1-((1*H*-benzimidazol-1-yl)methyl)-1*H*-imidazole ligands and two O atoms (O1 and O2) from two water molecules in equatorial positions, and by two terminal Cl atoms (Cl1 and Cl1A) in axial positions (Fig. 1). The benzimidazole and the imidazole moieties are nearly orthogonal to each other, with a dihedral angle of 84.27 (17) °. N—H···O, O—H···N, O—H···O and O—H···Cl hydrogen bonds (Table 2) between benzimidazole groups and solvent water molecules, between solvent water molecules and benzimidazole N atoms, between coordinating water molecules and solvent water molecules, between solvent water molecules and Cl atoms and between solvent water molecules and solvent water molecules of adjacent molecules consolidate the crystal packing (Fig. 2).

### Experimental

A mixture of CdCl_2_ (0.1 mmol), 1-((1*H*-benzimidazol-1-yl)methyl)-1*H*-imidazole (0.1 mmol) and water (10 ml) was placed in a 25 ml Teflon-lined stainless steel vessel and heated at 353 K for 72 h, then cooled to room temperature. Colourless crystals were obtained from the filtrate and dried in air.

### Refinement

H atoms bound to C and N atoms were positioned geometrically and refined as riding atoms, with C—H = 0.93 (aromatic) Å and 0.97 (CH_2_) Å, N—H = 0.86 Å. H atoms bound to O atoms were found from difference maps and refined with distance restraints of O—H = 0.85 Å. All H atoms were refined with U_iso_(H) = 1.2 U_eq_(C,N,O).

### Crystal data

**Table:**

[CdCl_2_(C_11_H_10_N_4_)_2_(H_2_O)_2_]·6H_2_O	*F*(000) = 740
*M**_r_* = 723.89	*D*_x_ = 1.583 Mg m^−^^3^
Monoclinic, *P*2/*c*	Mo *K*α radiation, λ = 0.71073 Å
*a* = 8.3562 (17) Å	Cell parameters from 4042 reflections
*b* = 10.236 (2) Å	θ = 2.0–27.9°
*c* = 17.972 (4) Å	µ = 0.95 mm^−^^1^
β = 98.80 (3)°	*T* = 293 K
*V* = 1519.1 (5) Å^3^	Prism, colorless
*Z* = 2	0.18 × 0.17 × 0.14 mm

### Data collection

**Table:**

Rigaku Saturn diffractometer	3632 independent reflections
Radiation source: fine-focus sealed tube	3345 reflections with *I* > 2σ(*I*)
Graphite monochromator	*R*_int_ = 0.044
Detector resolution: 28.5714 pixels mm^-1^	θ_max_ = 27.9°, θ_min_ = 2.3°
ω scans	*h* = −10→11
Absorption correction: multi-scan (*CrystalClear*; Rigaku/MSC, 2004)	*k* = −13→12
*T*_min_ = 0.847, *T*_max_ = 0.878	*l* = −23→23
18365 measured reflections	

### Refinement

**Table:**

Refinement on *F*^2^	Primary atom site location: structure-invariant direct methods
Least-squares matrix: full	Secondary atom site location: difference Fourier map
*R*[*F*^2^ > 2σ(*F*^2^)] = 0.047	Hydrogen site location: inferred from neighbouring sites
*wR*(*F*^2^) = 0.123	H-atom parameters constrained
*S* = 1.00	*w* = 1/[σ^2^(*F*_o_^2^) + (0.0656*P*)^2^ + 1.0048*P*] where *P* = (*F*_o_^2^ + 2*F*_c_^2^)/3
3632 reflections	(Δ/σ)_max_ < 0.001
188 parameters	Δρ_max_ = 0.66 e Å^−^^3^
0 restraints	Δρ_min_ = −0.63 e Å^−^^3^

### Special details

**Table:**

Geometry. All e.s.d.'s (except the e.s.d. in the dihedral angle between two l.s. planes) are estimated using the full covariance matrix. The cell e.s.d.'s are taken into account individually in the estimation of e.s.d.'s in distances, angles and torsion angles; correlations between e.s.d.'s in cell parameters are only used when they are defined by crystal symmetry. An approximate (isotropic) treatment of cell e.s.d.'s is used for estimating e.s.d.'s involving l.s. planes.
Refinement. Refinement of *F*^2^ against ALL reflections. The weighted *R*-factor *wR* and goodness of fit *S* are based on *F*^2^, conventional *R*-factors *R* are based on *F*, with *F* set to zero for negative *F*^2^. The threshold expression of *F*^2^ > σ(*F*^2^) is used only for calculating *R*-factors(gt) *etc*. and is not relevant to the choice of reflections for refinement. *R*-factors based on *F*^2^ are statistically about twice as large as those based on *F*, and *R*- factors based on ALL data will be even larger.

### Fractional atomic coordinates and isotropic or equivalent isotropic displacement parameters (Å^2^)

**Table:**

	*x*	*y*	*z*	*U*_iso_*/*U*_eq_	
Cd1	0.5000	0.48165 (3)	0.2500	0.03867 (14)	
Cl1	0.79282 (12)	0.51534 (10)	0.32722 (6)	0.0508 (2)	
N1	0.4065 (3)	0.4475 (3)	0.36168 (15)	0.0386 (6)	
N2	0.3973 (3)	0.3597 (3)	0.47262 (14)	0.0344 (6)	
N3	0.3091 (4)	0.0802 (3)	0.58812 (15)	0.0411 (6)	
H3A	0.3310	0.1032	0.6346	0.049*	
N4	0.2998 (3)	0.0876 (3)	0.46398 (14)	0.0393 (6)	
O1	0.5000	0.7124 (4)	0.2500	0.0972 (19)	
H1W	0.5785	0.7648	0.2488	0.117*	
O2	0.5000	0.2521 (4)	0.2500	0.0611 (11)	
H2W	0.4388	0.1999	0.2694	0.073*	
O3	0.0000	0.3013 (4)	0.2500	0.0536 (9)	
H3W	0.0609	0.3445	0.2252	0.064*	
O4	0.2544 (3)	0.1273 (2)	0.31049 (13)	0.0485 (6)	
H4W	0.2700	0.1245	0.3583	0.058*	
H5W	0.1619	0.1616	0.2969	0.058*	
O5	0.0000	0.7111 (4)	0.2500	0.0699 (12)	
H6W	0.0520	0.6677	0.2212	0.084*	
O6	0.2530 (4)	0.1123 (3)	0.74290 (16)	0.0614 (8)	
H7W	0.1698	0.1537	0.7519	0.074*	
H8W	0.2717	0.0525	0.7759	0.074*	
C1	0.4765 (4)	0.3664 (3)	0.41313 (18)	0.0380 (7)	
H1	0.5698	0.3192	0.4089	0.046*	
C2	0.2756 (4)	0.4962 (3)	0.3900 (2)	0.0401 (7)	
H2	0.2025	0.5568	0.3658	0.048*	
C3	0.2686 (4)	0.4432 (4)	0.45811 (19)	0.0417 (7)	
H3	0.1915	0.4601	0.4891	0.050*	
C4	0.4356 (4)	0.2751 (3)	0.53799 (18)	0.0419 (7)	
H4A	0.4080	0.3195	0.5820	0.050*	
H4B	0.5511	0.2581	0.5467	0.050*	
C5	0.3464 (4)	0.1478 (3)	0.52811 (17)	0.0361 (7)	
C6	0.2252 (4)	−0.0263 (3)	0.48364 (19)	0.0373 (7)	
C7	0.1457 (5)	−0.1248 (3)	0.4376 (2)	0.0470 (8)	
H7	0.1395	−0.1222	0.3855	0.056*	
C8	0.0773 (5)	−0.2257 (4)	0.4728 (2)	0.0511 (9)	
H8	0.0243	−0.2924	0.4437	0.061*	
C9	0.0854 (5)	−0.2303 (4)	0.5508 (2)	0.0517 (9)	
H9	0.0380	−0.3001	0.5724	0.062*	
C10	0.1616 (5)	−0.1345 (4)	0.5965 (2)	0.0503 (9)	
H10	0.1675	−0.1379	0.6486	0.060*	
C11	0.2299 (4)	−0.0316 (3)	0.56101 (19)	0.0385 (7)	

### Atomic displacement parameters (Å^2^)

**Table:**

	*U*^11^	*U*^22^	*U*^33^	*U*^12^	*U*^13^	*U*^23^
Cd1	0.0458 (2)	0.0382 (2)	0.0342 (2)	0.000	0.01327 (14)	0.000
Cl1	0.0476 (5)	0.0586 (6)	0.0476 (5)	−0.0031 (4)	0.0113 (4)	0.0018 (4)
N1	0.0438 (15)	0.0381 (15)	0.0355 (14)	−0.0018 (12)	0.0117 (12)	0.0001 (11)
N2	0.0388 (14)	0.0327 (14)	0.0328 (13)	−0.0046 (11)	0.0087 (11)	−0.0006 (10)
N3	0.0540 (17)	0.0415 (16)	0.0277 (12)	−0.0035 (13)	0.0059 (11)	0.0014 (11)
N4	0.0481 (16)	0.0393 (16)	0.0318 (13)	−0.0065 (12)	0.0099 (11)	−0.0001 (11)
O1	0.064 (3)	0.038 (2)	0.186 (6)	0.000	0.006 (3)	0.000
O2	0.081 (3)	0.0365 (19)	0.077 (3)	0.000	0.048 (2)	0.000
O3	0.058 (2)	0.051 (2)	0.054 (2)	0.000	0.0149 (18)	0.000
O4	0.0614 (16)	0.0493 (15)	0.0362 (12)	−0.0038 (12)	0.0122 (11)	0.0005 (11)
O5	0.069 (3)	0.052 (2)	0.098 (3)	0.000	0.039 (2)	0.000
O6	0.077 (2)	0.0509 (16)	0.0608 (17)	0.0045 (14)	0.0253 (15)	0.0083 (13)
C1	0.0393 (17)	0.0388 (17)	0.0383 (16)	0.0052 (13)	0.0131 (13)	0.0004 (13)
C2	0.0389 (18)	0.0393 (18)	0.0433 (19)	0.0060 (13)	0.0102 (14)	0.0026 (13)
C3	0.0403 (18)	0.0451 (19)	0.0435 (18)	0.0019 (14)	0.0181 (14)	−0.0022 (15)
C4	0.0484 (19)	0.0424 (18)	0.0337 (16)	−0.0060 (15)	0.0026 (14)	0.0012 (13)
C5	0.0410 (17)	0.0357 (17)	0.0324 (15)	0.0000 (13)	0.0086 (13)	0.0012 (13)
C6	0.0415 (17)	0.0351 (17)	0.0360 (16)	0.0015 (13)	0.0082 (13)	0.0045 (13)
C7	0.057 (2)	0.0396 (19)	0.0442 (19)	−0.0036 (16)	0.0091 (16)	−0.0046 (15)
C8	0.049 (2)	0.0362 (18)	0.066 (2)	−0.0021 (15)	0.0052 (18)	−0.0041 (17)
C9	0.050 (2)	0.0383 (19)	0.067 (2)	−0.0038 (16)	0.0099 (18)	0.0153 (17)
C10	0.059 (2)	0.046 (2)	0.0466 (19)	−0.0031 (17)	0.0098 (17)	0.0145 (16)
C11	0.0418 (18)	0.0367 (17)	0.0375 (17)	0.0007 (13)	0.0078 (14)	0.0051 (13)

### Geometric parameters (Å, º)

**Table:**

Cd1—N1	2.289 (3)	O5—H6W	0.8500
Cd1—N1^i^	2.289 (3)	O6—H7W	0.8499
Cd1—O2	2.349 (4)	O6—H8W	0.8500
Cd1—O1	2.362 (4)	C1—H1	0.9300
Cd1—Cl1	2.6445 (13)	C2—C3	1.349 (5)
Cd1—Cl1^i^	2.6445 (13)	C2—H2	0.9300
N1—C1	1.311 (4)	C3—H3	0.9300
N1—C2	1.369 (4)	C4—C5	1.498 (5)
N2—C1	1.343 (4)	C4—H4A	0.9700
N2—C3	1.367 (4)	C4—H4B	0.9700
N2—C4	1.455 (4)	C6—C11	1.386 (5)
N3—C5	1.357 (4)	C6—C7	1.405 (5)
N3—C11	1.373 (4)	C7—C8	1.380 (5)
N3—H3A	0.8600	C7—H7	0.9300
N4—C5	1.312 (4)	C8—C9	1.394 (6)
N4—C6	1.394 (4)	C8—H8	0.9300
O1—H1W	0.8500	C9—C10	1.372 (6)
O2—H2W	0.8501	C9—H9	0.9300
O3—H3W	0.8500	C10—C11	1.399 (5)
O4—H4W	0.8502	C10—H10	0.9300
O4—H5W	0.8499		
			
N1—Cd1—N1^i^	162.42 (14)	C3—C2—H2	125.1
N1—Cd1—O2	81.21 (7)	N1—C2—H2	125.1
N1^i^—Cd1—O2	81.21 (7)	C2—C3—N2	106.4 (3)
N1—Cd1—O1	98.79 (7)	C2—C3—H3	126.8
N1^i^—Cd1—O1	98.79 (7)	N2—C3—H3	126.8
O2—Cd1—O1	180.000 (1)	N2—C4—C5	112.2 (3)
N1—Cd1—Cl1	88.40 (8)	N2—C4—H4A	109.2
N1^i^—Cd1—Cl1	93.88 (8)	C5—C4—H4A	109.2
O2—Cd1—Cl1	97.49 (2)	N2—C4—H4B	109.2
O1—Cd1—Cl1	82.51 (2)	C5—C4—H4B	109.2
N1—Cd1—Cl1^i^	93.88 (8)	H4A—C4—H4B	107.9
N1^i^—Cd1—Cl1^i^	88.40 (8)	N4—C5—N3	112.7 (3)
O2—Cd1—Cl1^i^	97.49 (2)	N4—C5—C4	126.0 (3)
O1—Cd1—Cl1^i^	82.51 (2)	N3—C5—C4	121.3 (3)
Cl1—Cd1—Cl1^i^	165.01 (5)	C11—C6—N4	110.0 (3)
C1—N1—C2	105.3 (3)	C11—C6—C7	120.1 (3)
C1—N1—Cd1	122.7 (2)	N4—C6—C7	129.9 (3)
C2—N1—Cd1	132.0 (2)	C8—C7—C6	117.3 (3)
C1—N2—C3	106.6 (3)	C8—C7—H7	121.3
C1—N2—C4	126.8 (3)	C6—C7—H7	121.3
C3—N2—C4	126.6 (3)	C7—C8—C9	121.8 (4)
C5—N3—C11	107.4 (3)	C7—C8—H8	119.1
C5—N3—H3A	126.3	C9—C8—H8	119.1
C11—N3—H3A	126.3	C10—C9—C8	121.7 (3)
C5—N4—C6	104.7 (3)	C10—C9—H9	119.2
Cd1—O1—H1W	129.1	C8—C9—H9	119.2
Cd1—O2—H2W	129.0	C9—C10—C11	116.7 (3)
H4W—O4—H5W	107.3	C9—C10—H10	121.7
H7W—O6—H8W	107.2	C11—C10—H10	121.7
N1—C1—N2	111.9 (3)	N3—C11—C6	105.2 (3)
N1—C1—H1	124.1	N3—C11—C10	132.4 (3)
N2—C1—H1	124.1	C6—C11—C10	122.4 (3)
C3—C2—N1	109.8 (3)		
			
N1^i^—Cd1—N1—C1	48.6 (3)	C6—N4—C5—N3	0.8 (4)
O2—Cd1—N1—C1	48.6 (3)	C6—N4—C5—C4	179.0 (3)
O1—Cd1—N1—C1	−131.4 (3)	C11—N3—C5—N4	−1.0 (4)
Cl1—Cd1—N1—C1	−49.2 (3)	C11—N3—C5—C4	−179.4 (3)
Cl1^i^—Cd1—N1—C1	145.6 (3)	N2—C4—C5—N4	29.7 (5)
N1^i^—Cd1—N1—C2	−129.5 (3)	N2—C4—C5—N3	−152.2 (3)
O2—Cd1—N1—C2	−129.5 (3)	C5—N4—C6—C11	−0.3 (4)
O1—Cd1—N1—C2	50.5 (3)	C5—N4—C6—C7	176.5 (4)
Cl1—Cd1—N1—C2	132.7 (3)	C11—C6—C7—C8	−1.2 (5)
Cl1^i^—Cd1—N1—C2	−32.5 (3)	N4—C6—C7—C8	−177.8 (4)
C2—N1—C1—N2	0.4 (4)	C6—C7—C8—C9	0.2 (5)
Cd1—N1—C1—N2	−178.1 (2)	C7—C8—C9—C10	0.2 (6)
C3—N2—C1—N1	−0.5 (4)	C8—C9—C10—C11	0.3 (6)
C4—N2—C1—N1	176.6 (3)	C5—N3—C11—C6	0.8 (4)
C1—N1—C2—C3	−0.2 (4)	C5—N3—C11—C10	−178.5 (4)
Cd1—N1—C2—C3	178.1 (2)	N4—C6—C11—N3	−0.3 (4)
N1—C2—C3—N2	−0.1 (4)	C7—C6—C11—N3	−177.5 (3)
C1—N2—C3—C2	0.3 (4)	N4—C6—C11—C10	179.0 (3)
C4—N2—C3—C2	−176.7 (3)	C7—C6—C11—C10	1.8 (5)
C1—N2—C4—C5	−91.6 (4)	C9—C10—C11—N3	177.8 (4)
C3—N2—C4—C5	84.8 (4)	C9—C10—C11—C6	−1.4 (5)

Symmetry code: (i) −*x*+1, *y*, −*z*+1/2.

### Hydrogen-bond geometry (Å, º)

**Table:**

*D*—H···*A*	*D*—H	H···*A*	*D*···*A*	*D*—H···*A*
N3—H3*A*···O6	0.86	2.15	2.909 (4)	148
O2—H2*W*···O4	0.85	1.95	2.776 (3)	162
O4—H4*W*···N4	0.85	1.91	2.756 (4)	171
O4—H5*W*···O3	0.85	2.06	2.857 (4)	156
O3—H3*W*···Cl1^i^	0.85	2.41	3.234 (3)	165
O5—H6*W*···Cl1^i^	0.85	2.28	3.110 (3)	164
O1—H1*W*···O6^ii^	0.85	1.88	2.723 (4)	173
O6—H7*W*···O5^iii^	0.85	1.98	2.799 (4)	162
O6—H8*W*···O4^iv^	0.85	1.96	2.737 (4)	152

Symmetry codes: (i) −*x*+1, *y*, −*z*+1/2; (ii) −*x*+1, −*y*+1, −*z*+1; (iii) −*x*, −*y*+1, −*z*+1; (iv) *x*, −*y*, *z*+1/2.
